# Building the foundations for global data banking in digital phenotyping for mental health

**DOI:** 10.1038/s44277-026-00066-z

**Published:** 2026-07-13

**Authors:** Bridianne O’Dea, Samra Naz, Larisa T. McLoughlin, Alexis E. Whitton

**Affiliations:** 1 Flinders University Institute for Mental Health and Wellbeing, College of Human Sciences and Culture, Flinders University, Adelaide, SA 5042 Australia; 2 Black Dog Institute, Faculty of Medicine and Health, University of New South Wales, Sydney, NSW 2031 Australia

**Keywords:** Biomarkers, Psychiatric disorders

## Abstract

Mental illness is a leading cause of global disability, underscoring the urgent need for scalable, data-driven approaches to early identification and intervention. Passive sensing technologies in mobile and wearable devices enable continuous and unobtrusive measurement of behavioural and physiological signals that may be relevant to mental health. When translated into interpretable digital markers through digital phenotyping, these data hold significant promise for advancing our understanding of the onset, course, and treatment of mental disorders. However, achieving real-world clinical utility requires large-scale, harmonised, and ethically governed databanks that enable replication and generalisability for diverse populations. Informed by recent international initiatives, the academic literature, and our own expertise in digital phenotyping, this Perspective outlines four key priorities for advancing digital phenotyping databanks in depression and anxiety. First, ensuring data quality through standardisation and harmonisation is essential to comparability across studies and to prevent fragmentation. Second, ethical data stewardship demands hybrid consent models that combine the scalability of broad consent with the flexibility of dynamic consent, ensuring meaningful participant control as analytics evolve. Third, robust, privacy-preserving information governance co-created with people with Lived Experience is vital to maintain trust and prevent misuse, with federated learning and open-source pipelines offering promising technical pathways. Finally, the field must promote data reuse by reforming incentive structures, recognising databank-based scholarship, and investing in sustainable infrastructures that reward secondary analyses. Collectively, these priorities offer a pragmatic framework for building equitable, transparent, and scientifically robust digital mental health databanks. Implementing these recommendations will require sustained international collaboration among researchers, funders, institutions, and people with Lived Experience. By aligning scientific rigour with ethical responsibility, digital phenotyping databanks can become transformative tools for advancing the global understanding and treatment of mental illness.

## Lay Summary

Researchers are exploring how data from smartphones and wearables, such as movement, sleep, and phone use, might help detect and track depression and anxiety. This article outlines what is needed to build large, shared databanks of these data that are both useful and trustworthy, including better data standards, stronger privacy protections, greater participant control over data, and incentives for researchers to reuse data. International collaboration could accelerate the development of more effective ways to understand, detect and treat mental health problems across diverse communities.

**Fig. 1:**
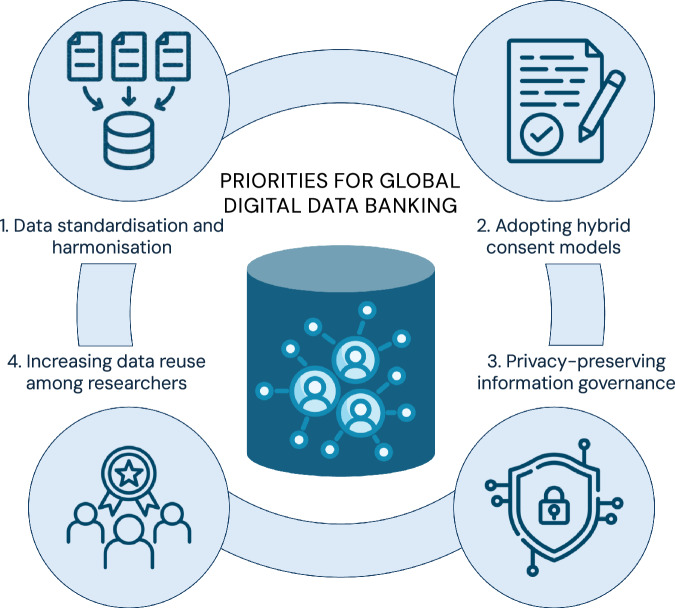
Priorities for Advancing Global Digital Phenotyping Data Banking.

Mental illness is a leading contributor to the global disease burden, with early identification and intervention critical for improving long-term outcomes. Passive sensing technologies, which unobtrusively capture behavioural and physiological data through mobile and wearable devices, and digital phenotyping, which converts raw sensor streams into behavioural signatures relevant to mental health, present new opportunities for addressing a range of mental health conditions [1, 2]. To achieve clinical impact, however, models must demonstrate validity, reliability, robustness and reproducibility across larger and more diverse samples and settings, to ensure scientific rigour and real-world applicability [3, 4]. Yet, there is a scarcity of suitable public datasets [5]. Reflecting this need, a recent Wellcome-Google workshop called for *‘large-scale mental health databanks incorporating passive sensing data’* to advance our understandings of the causes, consequences, and prevention of mental illness [6]. Further demonstrating this priority, Wellcome funded our team (Project PHONOTYPE) to establish one of the first digital phenotyping databanks focused on young people with depression [7]. However, building and sustaining this type of databank at scale raises significant methodological, ethical, and operational challenges that are widely recognised across the digital health field. Drawing on insights from the literature, lessons learned through our own digital phenotyping projects [8], and international workshops and scientific meetings convened or attended by our team, we synthesised these convergent challenges into four key priority areas to guide our approach to data banking. Shown in Fig. 1, these priorities reflect the domains in which we feel there is both substantial need and high potential for field-wide impact.

## Ensuring data quality through standardisation and harmonisation

Consistency and comparability of data in digital phenotyping studies are central to effective data banking. Standardisation (transforming data into uniform formats) and harmonisation (ensuring interoperability across datasets) are critical methodological challenges, with no consensus on protocols or core sets of harmonised features [4, 9]. Digital phenotyping data are often derived from multiple devices and sensor streams that differ in sampling rates, calibration, and signal fidelity. Synchronisation across devices, validation of sensor accuracy, proprietary algorithms, and handling of asynchronous and missing data introduce additional complexity and measurement variability that shape the quality and interpretability of downstream features. As such, understanding how data are generated is as critical as how they are standardised and stored. While technical guidelines for standardisation and harmonisation are essential and progressing across the field, maturation of these will take time, necessitating pragmatic approaches in the interim.

A trial of digital data stewardship models in the MindKind study found that transparent and thorough documentation on data collection, coding schemes, preprocessing, analysis code, data quality assessments and metadata for digital phenotyping studies can ensure current datasets are interpretable, reusable, and ready for future harmonisation efforts [10]. Harmonisation efforts in digital phenotyping can be further supported by researchers’ use of open science platforms with version control, hierarchical file ordering, and automated object identifier assignment. Similarly, open-source data processing pipelines, such as Cortex [11], may help researchers derive replicable clinical features from their digital phenotyping data and provide standardised reporting of data quality across studies.

For our Project PHONOTYPE databank, which is focused on young people with depression, we argue that initial standardisation and harmonisation efforts should prioritise mobility-sensor data (e.g. collected from GPS, network sensors, step counts), given that derived features (e.g. circadian rhythm, distance from home, location variance, number of locations visited, step count) have shown the strongest and most consistent associations with depressive symptoms in digital phenotyping studies to date [9]. This applied focus reflects both the strongest current empirical signals while providing a pragmatic starting point for developing transferable conventions across mental health conditions. These advances could be applied to support multimodal models for stratification, prediction, and personalised intervention of symptom domains and processes where motor and activity markers are highly relevant (e.g., fatigue, sleep disturbances, motivational impairments, psychomotor agitation or slowing). Ultimately, without deliberate efforts to address data standardisation and harmonisation, databanks risk becoming fragmented or underutilised.

## Adopting hybrid consent models

One of the central challenges for consent for digital data banking is that participants may not adequately comprehend the scope, granularity or potential future uses and processing of passive sensor data. This creates significant ethical risks, as almost any form of digital phenotyping data can constitute as sensitive information [12]. The application of novel analytical techniques, including those using generative artificial intelligence (AI), may make it difficult to remove individuals’ data from trained models should they withdraw consent. Conventional research consent protocols do not always accommodate the specialised requirements of digital phenotyping, and legal and ethical obligations vary across jurisdictions [13, 14]. Participants may not distinguish between consent to participate in research and consent to process personal data, leading to a ‘consent misconception’ [14]. Addressing this issue requires researchers to recognise the strong scientific, methodological, ethical, and legal rationale for distinguishing consent for data use from consent for data processing, rather than relying exclusively on conventional research standards.

With this in mind, digital phenotyping researchers may wish to adopt hybrid consent models that combine elements of dynamic consent with a ‘broad but deep’ approach [15]. Unlike dynamic consent’s study-by-study reconsent process, which risks decision fatigue and may slow large-scale phenotyping, ‘broad but deep’ consent involves a one-time, overarching authorisation for data use and processing covering the scope of an initial research proposal. This model supports scalability, maintains participant autonomy through structured re-engagement, and adapts to evolving values without overwhelming participants. In contexts where passive data are collected continuously, participants provide explicit consent at enrolment using tiered permissions for data banking. The databank operates under a governance framework that specifies data deidentification and use, security protocols and access approvals (including cross-site and international collaborations), permissible research domains and conditions (covering current and downstream uses), institutional oversight, and procedures for handling incidental findings. Researchers are then required to provide annual project summaries, transparently report emergent privacy risks, and issue opt-out consent updates only when project scope or access approvals change [15].

In our proposed hybrid consent approach, any changes or expansion to data processing (e.g., use of data in the development or application of new AI models within or across research groups) are to be managed through dynamic consent. This allows participants to update, pause, or withdraw permissions as analytics, collaborations, and cross-site projects evolve, thereby retaining meaningful control over their data [16, 17]. An extension of this model may involve greater participant involvement in authorising specific data processing activities, particularly where analyses introduce novel risks or deviate from the original research scope. At consent, participants can choose to contribute (or withdraw) their data from new model training while allowing past models to remain, with data already incorporated in completed analyses or trained models not removed. Researchers maintain transparency and reproducibility by using versioning and model lineage to document each model, including the training datasets, timepoints, consent status, and model locking to preserve its performance.

Reviews of dynamic consent in biobanking report substantial benefits, including greater participant engagement and retention [18]. Illustrative examples include the mPower study [19], which used a digital, interactive, tiered consent process with comprehension checks and granular sharing choices to empower participants and maximise data reuse, and the CHRIS biobank [20], which employed an online platform enabling granular consent revisions over time. Few changes were made in practice, but participants valued opportunities to do so. The adoption of a ‘broad but deep’ consent model is likely to be accepted by participants, given its use in existing epidemiological and biobank initiatives. Co-designed decision-making frameworks between researchers and participants may further strengthen trust and align data use with participant values, although the feasibility and scalability of such approaches require further empirical evaluation. Successful implementation of these approaches also requires clear consent materials, transparent communication, thorough planning, and robust technical reinforcement.

## Privacy-preserving Information Governance

Information governance refers to the physical, technical, and procedural measures that safeguard and support a data utility. Effective information governance defines custodianship, establishes processes for reviewing data use proposals, controls and authorises access, secures storage and transfer, maintains accurate record linkages, anonymises and encrypts data to prevent re-identification, manages disclosure risk, and ensures compliance with legal standards such as the right to erasure and misuse protections. The sensitive nature of digital phenotyping heightens these challenges, as breaches may expose participants, and broader groups who share demographic or behavioural characteristics, to stigma, profiling, discrimination, surveillance and targeted marketing, concerns that are well documented [10, 21, 22]. Strong information governance in digital databanks is therefore essential to protect participants’ privacy, uphold the rights of individuals, and maintain public trust.

Across 69 biobanks, Gille and colleagues [23] identified six key governance elements: communication, compliance, expert advice, external review, internal procedures, and partnerships. Notwithstanding these principles, most existing databanks are institutionally-led, with governance largely centred on academic or organisational control. However, emerging scholarship increasingly advocates for more participatory and democratic models of data governance. The ‘data-owning democracy’ proposes a two-layered approach to data stewardship, comprising infrastructure that enables citizens to collectively generate and govern digital data, alongside mechanisms that provide individuals with machine-readable access to their own data as it is produced [24]. The emphasis on participant empowerment highlights alternative custodial arrangements for databanks, in which individuals are able to ‘shield and share’ their data in ways that reflect their preferences and values. Within this context, community-governed or participant-led databanks may redistribute decision-making authority and align data use with participant priorities. In such models, researchers function primarily as data contributors and applicants, rather than custodians.

Building on this shift, we argue that databank governance for Project PHONOTYPE should prioritise (1) co-creation with Lived Experience experts and (2) privacy-preserving analytics. In practice, effective co-creation involves embedding Lived Experience experts as voting members on data access committees and governance boards, and co-authors of ethics protocols. Secondary data requests should include plain-language impact statements and justifications of participant benefit, alongside capacity-building initiatives that enable Lived Experience experts to engage with data features and analysis pipelines. Lived Experience experts can be involved in reviewing and ranking secondary data use priorities, co-develop principles for commercial partnerships, and to identify unacceptable use cases. For example, the MindKind project co-designed four governance and consent models with young people [10]. A subsequent randomised trial showed that while enrolment rates did not differ between models, clear youth-friendly explanations improved participation. Participants preferred culturally tailored policies that prohibited exploitative commercial use, ensured equitable access, and combined central oversight with local expertise to avoid health inequities and loss of trust. MindKind demonstrated that co-designed governance frameworks can improve transparency, build trust, and align governance with the values of diverse youth.

Privacy-preserving data analytics that do not require raw data sharing are also paramount to realising the scientific and clinical potential of digital phenotyping for mental health. Federated Learning (FL) is a distributed machine learning approach that can enable collaborative model training without exposing local data, reducing the risk of participant re-identification. FL has been applied to several types of health data, although, debate continues over its utility and trade-offs [25, 26]. While FL may offer enhanced privacy protections, improved regulatory and jurisdictional compliance across international research collaborations, increased participant trust, and greater scalability of research efforts, the approach has constraints. These include increased statistical complexity due to heterogeneous and sparse data, the need for stable network infrastructure, stringent communication protocols and end-to-end encryption as well as transparent governance and oversight. The technical and operational requirements inherent to FL can constrain flexibility for early-stage exploratory analyses and may create false reassurance about privacy. Accordingly, FL should be considered as one component of data banking architecture rather than a universal solution.

Emerging work suggests that ‘smart contracts’, defined as computationally coded agreements automatically executed under predefined conditions, may strengthen consent protections within FL [27]. By embedding individual-level consent preferences directly within data execution environments, smart contracts may help ensure that data are accessed only when contractual conditions are met. Collaboration among researchers, legal and technical specialists, ethicists and Lived Experience experts is needed to co-design and evaluate the acceptability, technical feasibility, and effectiveness for enforcing individual-level consent preferences in digital phenotyping research. Alternatively, open-source platforms and ‘data pipelines’ (e.g. Open mHealth, Cortex) can provide infrastructure for digital phenotyping analysis without centralising it. Ultimately, the success of digital mental health data banking will depend on the development of secure and privacy-preserving analytic infrastructures.

Another critical consideration for data banking is the complexity of international data governance. Digital phenotyping databanks operating across jurisdictions must navigate divergent legal and regulatory frameworks governing data protection, consent, and cross-border data transfer, making alignment challenging but essential for global collaboration. This requires harmonised governance standards, interoperable agreements, and ongoing policy coordination to ensure compliance while preserving the scientific value of international data banking. Emerging data architectures may shift from traditional data sharing toward query-based models, where researchers submit analytical questions executed within secure environments and receive aggregated outputs rather than raw data. Such approaches, potentially governed by smart contracts or automated compliance layers, could reduce privacy and governance burdens. However, these systems also introduce new considerations related to transparency, auditability, and methodological flexibility, and remain an important area for further development.

## Increasing data reuse among researchers

Ensuring the scientific and clinical value of databanks requires more than large-scale data storage. Although the benefits of open data are increasingly recognised, substantial gaps remain in researchers’ readiness and practices for data sharing. While the factors influencing data sharing and reuse science are multi-faceted and complex, commonly cited barriers include lack of rewards and formal recognition, resource and infrastructure constraints, lack of policies targeting reuse specifically, research culture and perceived norms [28, 29]. To maximise impact, investment in databanks must therefore be accompanied by systems-level reforms that actively promote data reuse among mental health researchers using both rewards and penalties.

Dedicated funding and award schemes for secondary data analysis and high-impact reuse could encourage wider uptake of archived datasets. Funding that prioritises scientific discovery through data reuse (e.g., Wellcome Trust’s Mental Health Award in Leveraging Longitudinal Data, United Kingdom Research and Innovation/Economic and Social Research Council’s secondary data analysis round), supports data infrastructure, and provides ongoing salaries for data custodians may shift the risk–reward balance in favour of reuse. Similarly, dedicated cash rewards, like the NIH Data Sharing Index (S-index) Challenge, may also help promote data sharing and reward exemplary data reuse. Such mechanisms are particularly important for Early Career Researchers working in resource-limited and competitive environments, enabling them to transform existing datasets into influential outputs. To further incentivise data reuse, research funders can also reframe data reuse as equivalent to novel data collection by mandating data reuse plans in grant applications and incorporating these as scored criteria [29].

Broader institutional reforms are also necessary. Research excellence and reward systems should formally recognise databank-based scholarship by incorporating reuse metrics into research tracking systems, thereby normalising secondary analysis [30]. Journals could further incentivise reuse by expediting peer review or waiving article processing fees for secondary analysis. Databanks themselves can also improve re-use by creating supportive review structures, such as the MindKind Project’s Data Usability Advisory Group, which provides feedback on data reuse proposals. By integrating these approaches, funders, publishers, and institutions can foster a sustainable ecosystem in which data reuse is feasible, incentivised, and professionally advantageous.

### Concluding remarks

Digital phenotyping data banking presents a transformative opportunity to strengthen the understanding of mental health across diverse populations. The priorities outlined here provide some of the foundations for building equitable, scalable, and trustworthy digital mental health databanks within and beyond our own research group. An ideal digital mental health databank would integrate several core features: standardised and well-documented multimodal data pipelines; hybrid, participant-centred consent models; privacy-preserving analytical infrastructure; and governance frameworks co-designed with people with Lived Experience and adaptable across jurisdictions. In practice, implementing such a system requires trade-offs between scalability, flexibility, privacy, and usability. Recognising and transparently managing these trade-offs will be essential for developing databanks that are both scientifically valuable and socially acceptable. However, achieving this vision will require global collaboration and ongoing deliberation between digital phenotyping researchers, Lived Experience experts, funders, publishers, and institutions. Future structured consensus-building efforts will be essential to refine and prioritise these directions, helping to ensure that digital mental health databanks are scientifically valuable resources for global mental health research.
